# Offloading operation bivariate extreme response statistics for FPSO vessel

**DOI:** 10.1038/s41598-023-31533-8

**Published:** 2023-03-22

**Authors:** Oleg Gaidai, Yu Cao, Xiaosen Xu, Yihan Xing

**Affiliations:** 1 Shanghai Ocean University, Shanghai, China; 2 Jiangsu University of Science and Technology, Zhenjiang, China; 3 University of Stavanger, Stavanger, Norway

**Keywords:** Civil engineering, Energy infrastructure, Mechanical engineering

## Abstract

The Floating Production Storage and Offloading unit (FPSO) is an offshore unit producing and storing crude oil prior to tanker transport. An important design concern is an accurate prediction of risky dynamic hawser tensions during FPSO offloading operations. Bivariate extreme hawser tension contours are important for selecting proper design values. This paper employed the AQWA hydrodynamic software to analyze vessel hydrodynamic wave loads dynamic response, acting on FPSO vessels under realistic sea state conditions. This paper presents an efficient method for estimating FPSO bivariate response statistics based on numerical simulations validated by various experiments. The bivariate Average Conditional Exceedance Rate (ACER2D) method offers an accurate bivariate extreme value probability distribution and return period contours estimation, utilizing available data efficiently. The two-dimensional probability contours, corresponding to low probability return periods, are easily obtained by the ACER2D method. The performance of the presented method has shown that the ACER2D method provides an efficient and accurate prediction of extreme return period contours. The suggested approach may be used for FPSO vessel design, minimizing potential FPSO hawser damage. Bivariate contours yield bivariate design points, as opposed to a pair of uncoupled univariate design points with the same return period as currently adopted in the industry.

## Introduction

FPSO is one of the most popular offshore units for the modern offshore energy industry. FPSO plays a significant role in offshore oil production because of its flexible seakeeping.

Offloading operations are indispensable during modern offshore energy production, thus being critical for FPSO design. There are two basic offloading interaction systems between FPSO and its shuttle tanker: the SBS (side-by-side) offloading; the tandem offloading. FPSO being permanently moored using either turret mooring or spread mooring system. Mooring hawsers are being utilized to interconnect FPSO and shuttle tanker to avoid drifting apart, and fenders are being located between the vessel gap to avoid collision during SBS offloading operation. Relative motions between two vessels, fenders' reaction forces and hawsers tensions are key issues during SBS offloading operation process. SBS offloading dynamic system failure may result in economic and environmental losses. In Ref.^1^, authors considered second-order SBS forces and moments according to the Wigley model. In Ref.^2^, time-domain simulation was used to assess the hydrodynamic response of LNG-FPSO with moored Liquid Natural Gas (LNG) vessels. In Ref.^3^, the authors studied SBS hydrodynamic interaction between LNG and FPSO by utilizing the linearized 3D potential method. Reference^4^ presented FPSO offloading operation reliability study; in Ref.^5^, SBS offloading system failure mode was highlighted. A hydrodynamic experimental study of an FPSO in the Gulf of Mexico deep-water region is presented in Ref.^6^. Reference^7^ studied the hydrodynamic performance of spread moored FPSO and shuttle tanker during SBS offloading operation. Reference^8^ proposed a novel anti-motion structure with a taper angle decreasing cylindrical FPSO dynamic response. Reference^9^ authors used response-based analysis for metocean design conditions for FPSO vessels off North West Shelf. Reference^10^ presented cargo transfer vessel offloading operations in the Libra field. Reference^11^ studied parameter optimization for an FPSO using a combination of improved fruit fly optimization and a back-propagation neural network. In this paper, AQWA commercial software, was employed to simulate a numerically dynamic offloading operation process between FPSO and shuttle tanker in frequency and time domains.

**Figure 1:**
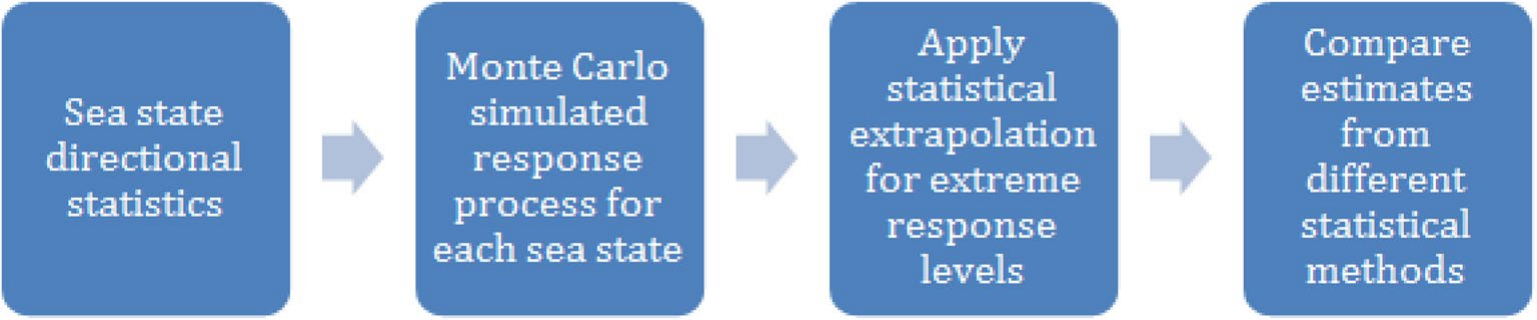
Flow chart for the described long-term statistical analysis.

In this study, authors employed a general Monte Carlo (MC) based technique, able to tackle inherent non-linear effects without significant simplifications except those inherent in the numerical model itself. The target bivariate extreme response distribution was obtained by combining MC simulations with the ACER2D extrapolation method^12–16^. Unlike univariate statistical techniques, the ACER2D method accounts for bivariate response correlation. The bivariate ACER2D approach is not the only technique to assess bivariate statistical distributions; see Ref.^16^ for the inverse first-order reliability method (IFORM) and Ref.^17^ for the second-order reliability method (SORM). Figure 1 presents a schematic flow chart for the MC-based long-term statistical analysis. In this study, bivariate extrapolation was performed instead of a conventional univariate one.

The main objective of this study is to advocate a bivariate statistical approach instead of a de-coupled univariate one.

## Wave statistics

**Figure 2:**
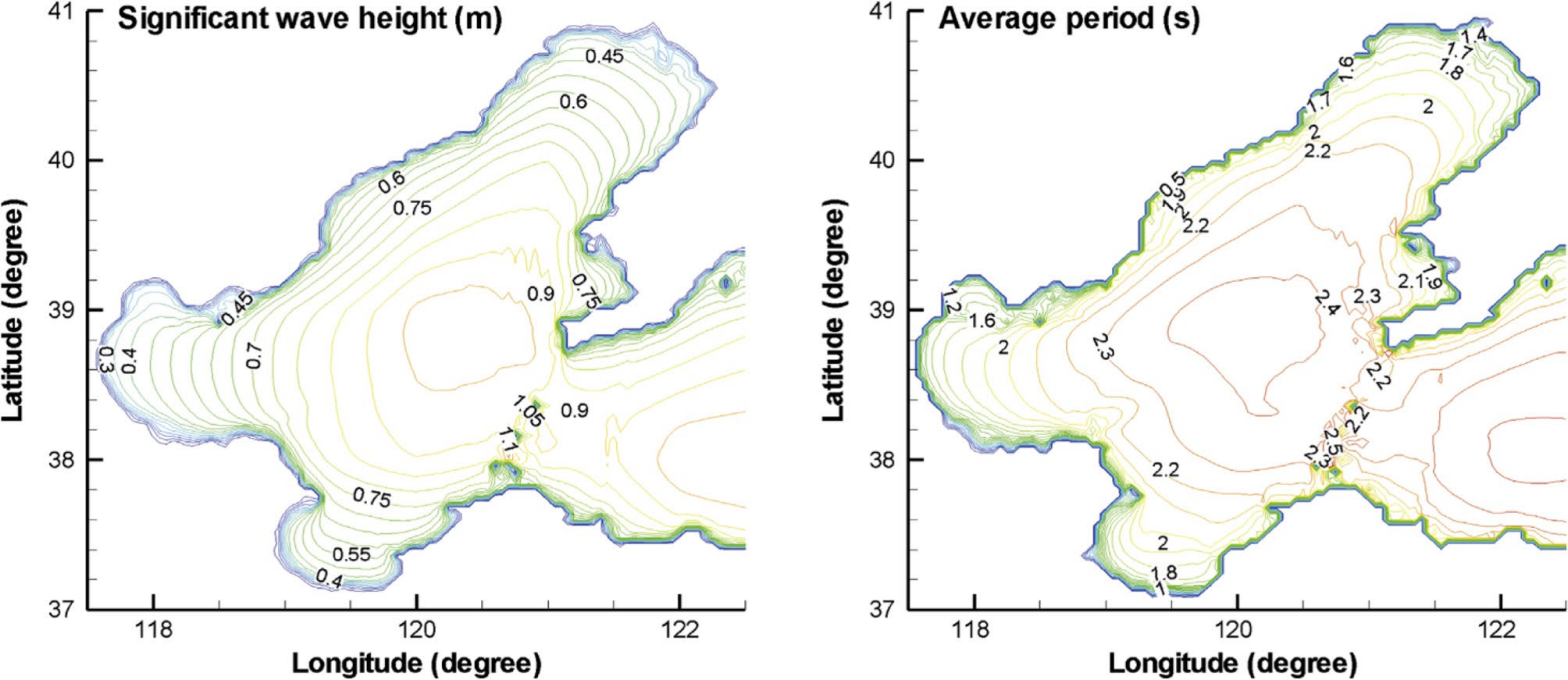
Annually averaged spatial distribution of wave height and period in the Bohai Sea^22^.

Satellite-based global wave statistics have been used to obtain a wave scatter diagram in the Bohai bay area. Global wave statistics based on satellite measurements were utilized to estimate wave scatter diagrams in relevant FPSO operational Bohai Bay in situ area; Global Wave Statistics Online^18^ data was utilized. Figure 2 presents the annually averaged spatial distribution of wave height and period in the Bohai Sea in situ area^7,17,19–23^.

Each random stationary sea state is specified by a JONSWAP wave spectrum, being one-sided power spectral density (PSD) ${S}_{\eta }^{+}\left(\omega \right)$ of wave elevation function $\eta (t)$, given $\omega >0$

1 $${S}_{\eta }^{+}\left(\omega \right)=\frac{\alpha {g}^{2}}{{\omega }^{5}}{\text{exp}}\left\{-\frac{5}{4}{\left(\frac{{\omega }_{p}}{\omega }\right)}^{4}+{\text{ln}}\gamma \cdot {\text{exp}}\left[-\frac{1}{2{\sigma }^{2}}{\left(\frac{\omega }{{\omega }_{p}}-1\right)}^{2}\right]\right\},$$

with $g=9.81$ m/s^2^, ${\omega }_{p}$ being the peak frequency in rad/s; and $\alpha$, $\gamma$ and $\sigma$ being the spectral shape parameters;$\sigma =0.07$ when $\omega \le {\omega }_{p}$; and $\sigma =0.09$ when $\omega >{\omega }_{p}$. For Bohai Bay relevant area, the sea state parameter $\gamma$ was chosen equal to 3.3; the parameter $\alpha$ determined from empirical relationship $\alpha =5.06{\left(\frac{{H}_{s}}{{T}_{p}}\right)}^{2}\left(1-0.287\cdot {\text{ln}}\gamma \right)$, see DNV rules^24,25^, with ${H}_{s}$ being the significant wave height, ${T}_{p}=\frac{2\pi }{{\omega }_{p}}$ being the spectral peak wave period.

## FPSO and shuttle tanker SBS operation particulars

**Figure 3:**
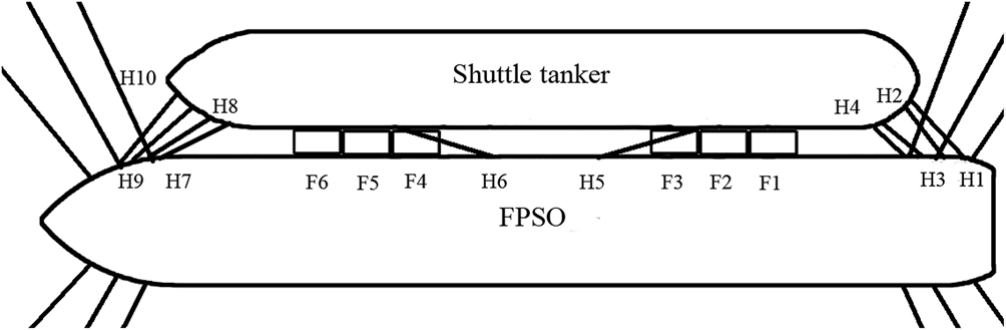
The basic layout of the SBS offloading along with the multi-point mooring system.

Table 1 presents FPSO and shuttle tanker parameters. FPSO was modelled as being moored with a spread mooring system. 12 anchor lines were placed between the FPSO stern and bow. The spread mooring system and SBS offloading system, were presented in Fig. 2. Figure 3 shows fenders F1 to F6 and hawsers H1 to H10.

**Table 1:** Some details of FPSO vessel and shuttle tanker (numbers have been rounded off).

Designation	Signal	Unit	FPSO	Shuttle tanker
Overall length	*L*_*OA*_	m	236	207
Length between perpendiculars	*L*_*PP*_	m	225	194
Breadth	*B*	m	46	36
Depth	*D*	m	24	16
Draft	*T*	m	11	9
Displacement	∆	t	104,748	49,444
Above base center of gravity	*KG*	m	10	12
Centre of gravity	*L*_*CG*_	m	110	99

For the bivariate statistical analysis in this paper, hawsers H1 and H2, located at the FPSO stern, see Fig. 3, have been chosen. There is a clear correlation between H1 and H2 tensions. Ten hawsers were made of nylon ropes having 110 mm diameter, connecting FPSO with its shuttle tanker. The fender friction coefficient $\mu$ was set equal to 0.2.

**Figure 4:**
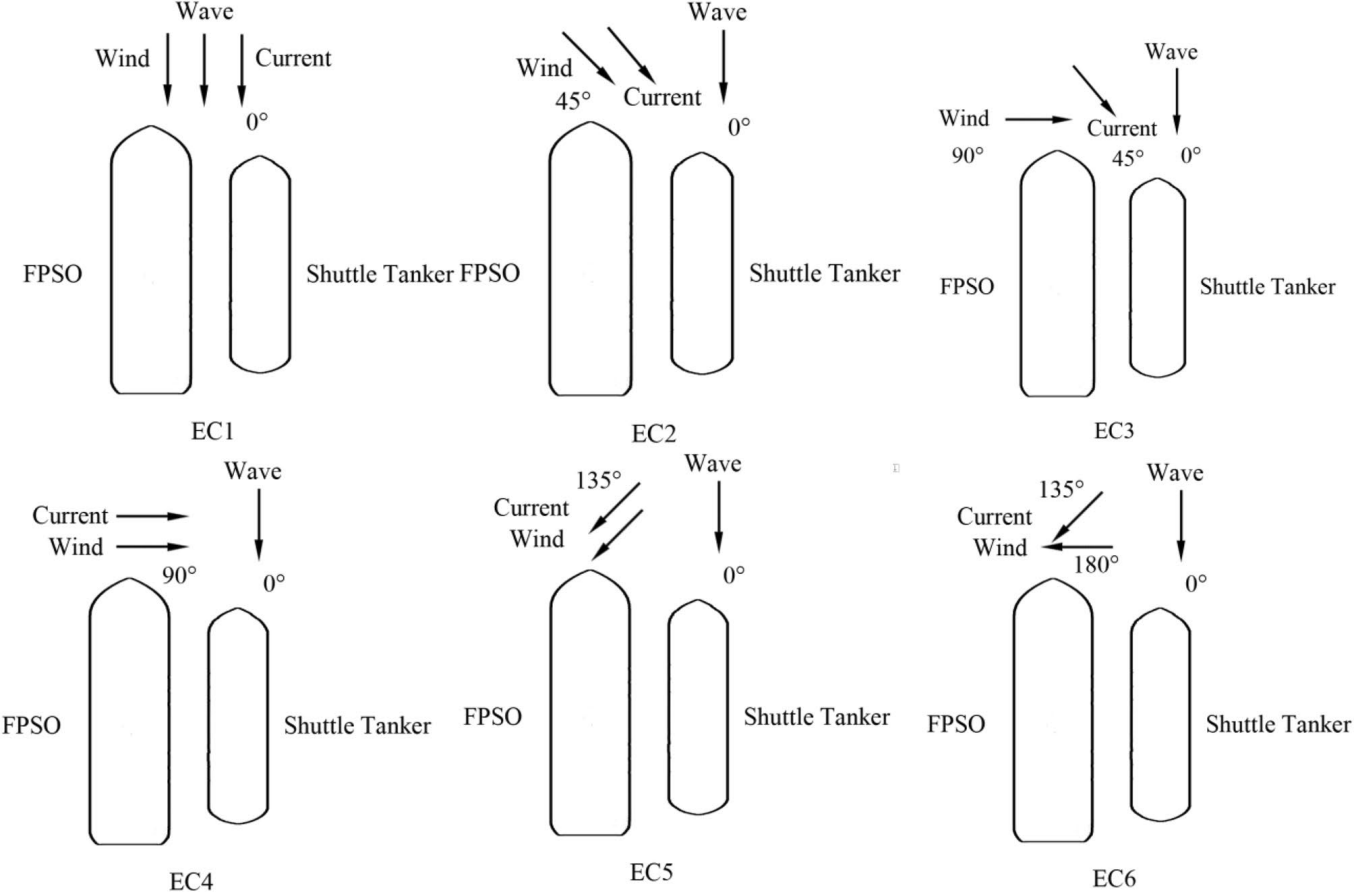
Six typical environmental conditions.

Figure 4 shows 6 sea environmental conditions and direction combinations of winds, waves, and currents for chosen environmental conditions. According to the American Petroleum Institute (API) reference rules^26,27^ for the multi-point mooring system and SBS offloading system, the SBS offloading operation is restricted only for head sea conditions with significant wave height ${H}_{s}$ equals 2.4 m. The spectrum peak period for the JONSWAP spectrum is 8.0 s and the peak enhancement factor is 1.9. Therefore, several typical environmental conditions are selected for calculation, see Fig. 4.

## Numerical simulation results

**Figure 5:**
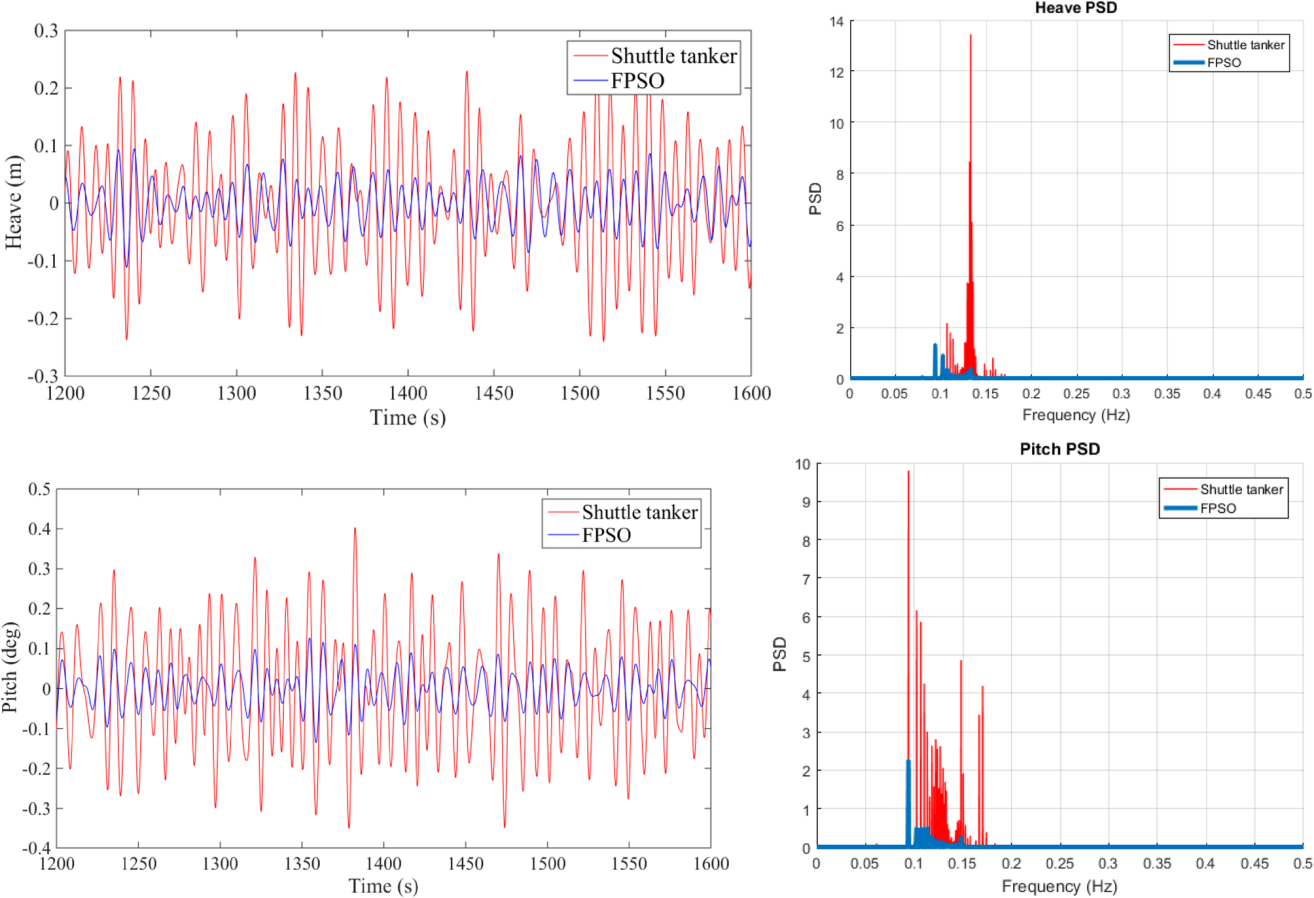
Vessel sectional time series data (left), power spectral densities (right), PSD units in m^2^/Hz (heave) and rad^2^/Hz (pitch).

**Figure 6:**
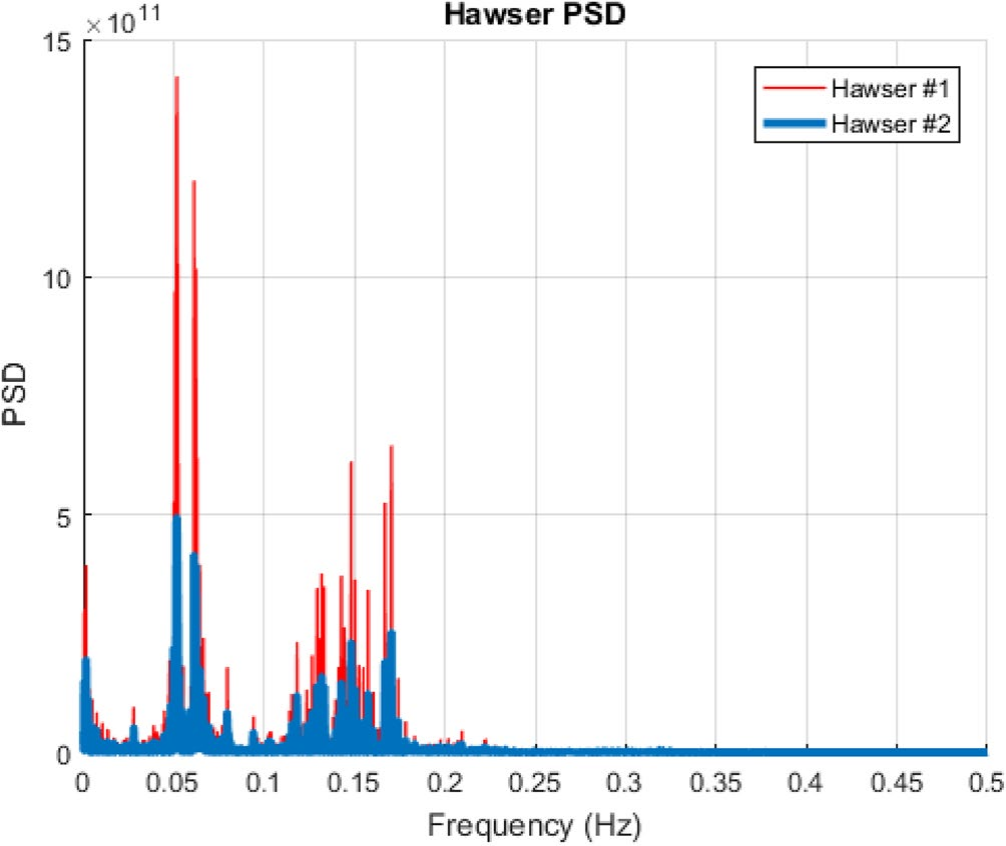
Selected hawser tension PSD, dimensional units N^2^/Hz.

AQWA time-domain simulation method was adopted in the time-domain analysis to simulate vessel motions under non-linear forces acting on hawsers and fenders. Duration of each simulation was set to three hours with six different seeds and the time step is 0.2 s. Due to initial transient effect, the first one-hour simulation data was removed in numerical analysis. Figure 5 presents sectional times series data and power spectral densities (PSD) in EC1. This section briefly highlights frequency and time domain numerical results^27–29^ (Fig. 6).

**Figure 7:**
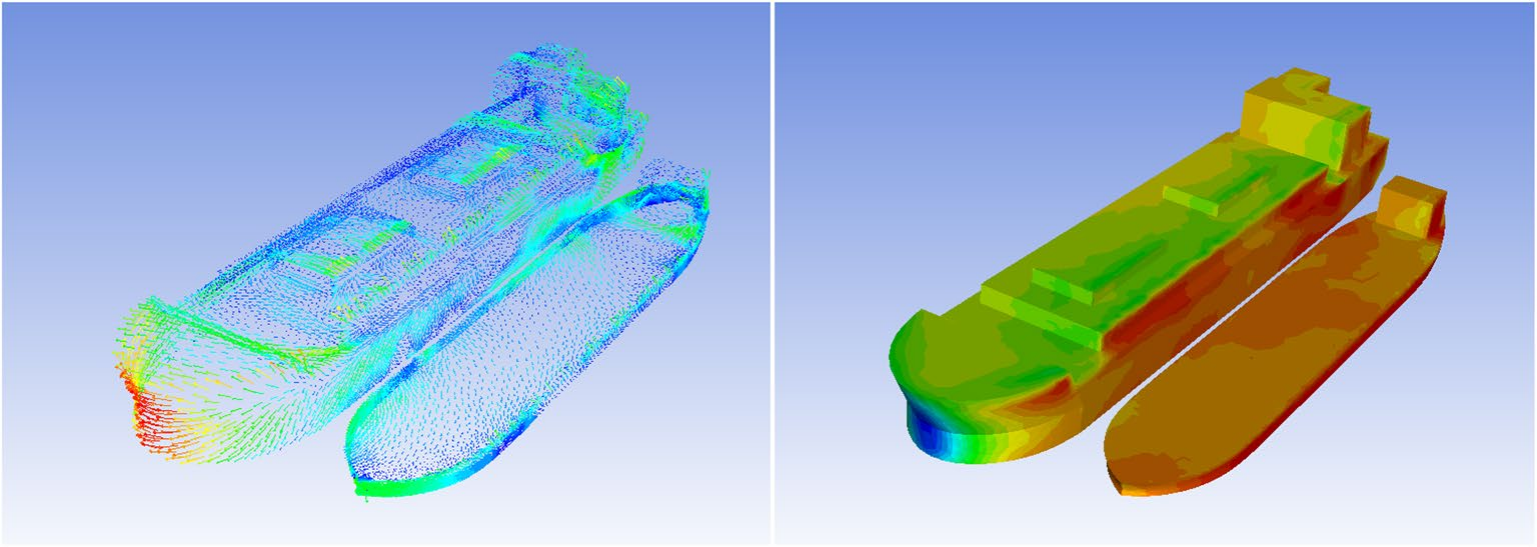
Example of the velocity vector field along with pressure distribution^20^.

Figure 7 presents the PSD of the tension of H1 and H2, indicating that more energy is observed for lower frequencies due to SBS offloading system.

## Experimental validation

Force due to wind and current were assessed by using ANSYS Fluent software. Lateral force, longitudinal force, moment about vertical z-axis have been assessed. Numerical non-dimensionalized force as well as moment coefficients for a given FPSO vessel have been validated, using data from Refs.^30,31^.

Figure 7 gives an example numerically simulated of velocity vector field, along with pressure distribution^20,21^. The latter numerical simulation has been used for validation of above discussed experimental results. In this computational fluid dynamics (CFD) simulation wind and currents effects have been taken into account.

**Figure 8:**
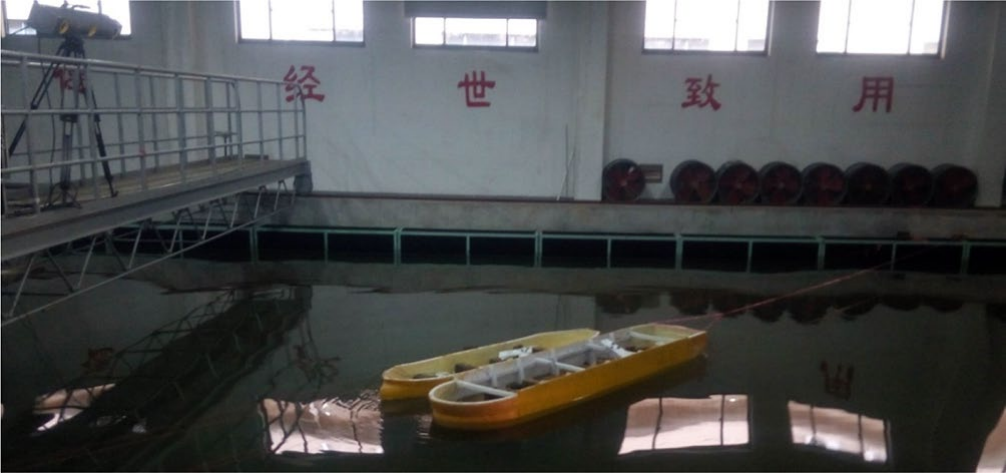
FPSO along with shuttle tanker model; side-by-side mooring being installed.

Lab experiment was carried out in the Jiangsu University of Science and Technology (China) towing tank, having model scale of 1:80; an equivalent truncated mooring system has been adopted. References^30,31^ give instructions for the truncated mooring system. Two vessel response-amplitude-operator (RAO) has been obtained, given excitations due to regular waves with a given spectrum. Figure 8 presents laboratory experimental setup.

**Figure 9:**
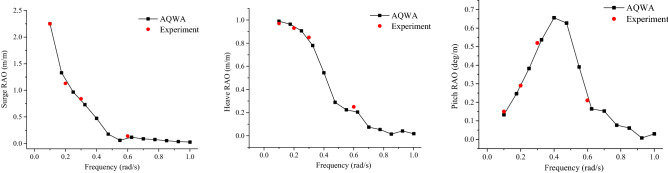
Measured and calculated FPSO motion RAOs (head sea).

RAOs for the FPSO were calculated from AQWA and then have been compared with available experimental results, see Fig. 9. These results indicated that numerical results from AQWA were in good agreement with experimental data, thus validating numerical AQWA results.

CFD non-dimensionalized wind and drag force along with single FPSO moment coefficients have beee validated by available experimental data from Refs.^30,31^.

## The ACER2D method: a statistical approach

The bivariate (2D) Average Conditional Exceedance Rate, briefly ACER2D, was applied to analyse FPSO hawser tensions to study extreme bivariate statistics. One advantage of ACER2D is its ability to account for the clustering effect of response peaks within extreme value statistics. The clustering effect is important for the case studied in this paper since hawser tensions possess distinct narrow-band effects, as seen in Fig. 7. Note that both stochastic response processes (H1 and H2 FPSO stern hawsers in this paper) are synchronous in time; the latter is beneficial for studying coupling effects and bivariate statistics. A brief introduction of the bivariate ACER2D method is given below^19,32^. See Refs.^16,33^ for alternative statistical and reliability approaches.

This paper considers bivariate random process $Z(t)=(X(t),Y(t))$, with two scalar processes $X(t),Y(t)$, either simulated or measured synchronously, over a period of time $(0,T)$. The data sample $({X}_{1},{Y}_{1}),\dots ,({X}_{N},{Y}_{N})$ is assumed to be recorded at *N* time equidistant instants ${t}_{1},\dots ,{t}_{N}$ given observation time span $(0,T)$.

Next, let us estimate the CDF (joint cumulative distribution function) $P\left(\xi ,\eta \right):=\mathrm{ Prob }\left({\widehat{X}}_{N}\le \xi ,{\widehat{Y}}_{N}\le \eta \right)$ of the maxima vector $\left({\widehat{X}}_{N},{\widehat{Y}}_{N}\right)$, with ${\widehat{X}}_{N}=\mathrm{max}\left\{{X}_{j} ;j=1,\dots ,N\right\}$, and ${\widehat{Y}}_{N}=\mathrm{max}\left\{{Y}_{j} ;j=1,\dots ,N\right\}$. In this paper $\xi$ and $\eta$ being H_1_ and H_2_ hawser tensions correspondingly. The non-exceedance event is identified: ${\mathcal{C}}_{kj}\left(\xi ,\eta \right):=\{{X}_{j-1}\le \xi ,{Y}_{j-1}\le \eta ,\dots ,{X}_{j-k+1}\le \xi ,{Y}_{j-k+1}\le \eta \}$ for $1\le k\le j\le N+1$. According to the definition of CDF $P(\xi ,\eta )$

2 $$\begin{array}{ll}& P(\xi ,\eta )=\mathrm{ Prob }({\mathcal{C}}_{N+1,N+1}(\xi ,\eta ))\\ & =\mathrm{ Prob }({X}_{N}\le \xi ,{Y}_{N}\le \eta | {\mathcal{C}}_{NN}(\xi ,\eta ))\cdot \mathrm{Prob }({\mathcal{C}}_{NN}(\xi ,\eta ))\\ & =\prod_{j=2}^{N}\mathrm{ Prob }({X}_{j}\le \xi ,{Y}_{j}\le \eta | {\mathcal{C}}_{jj}(\xi ,\eta ))\cdot \mathrm{Prob }({\mathcal{C}}_{22}(\xi ,\eta )).\end{array}$$

The CDF $P(\xi ,\eta )$ may be represented as in Refs.^7,8,34–36^

3 $$P\left(\xi ,\eta \right)\approx \mathrm{exp}\left\{-\sum_{j=k}^{N}\left({\alpha }_{kj}\left(\xi ;\eta \right)+{\beta }_{kj}\left(\eta ;\xi \right)-{\gamma }_{kj}\left(\xi ,\eta \right)\right)\right\},\mathrm{ for large }\xi \mathrm{and }\eta ,$$

for a large conditioning parameter $k$ with ${\alpha }_{kj}\left(\xi ;\eta \right) : = \text{ Prob }({X}_{j}>\xi | {\mathcal{C}}_{kj}(\xi ,\eta )), {\beta }_{kj}\left(\eta ;\xi \right) : = \text{Prob }({Y}_{j}>\eta \left|{\mathcal{C}}_{kj}\left(\xi ,\eta \right)\right), {\gamma }_{kj}\left(\xi ,\eta \right) : = \text{Prob }({X}_{j}>\xi ,{Y}_{j}>\eta | {\mathcal{C}}_{kj}(\xi ,\eta ))$. Next, the $k$-th order bivariate ACER2D functions are defined

4 $${\mathcal{E}}_{k}\left(\xi ,\eta \right)= \frac{1}{N-k+1} \sum_{j=k}^{N}\left({\alpha }_{kj}\left(\xi ;\eta \right)+{\beta }_{kj}\left(\eta ;\xi \right)-{\gamma }_{kj}\left(\xi ,\eta \right)\right), k=1, 2,\dots$$

Then, when $$N" k$$

5 $$P\left(\xi ,\eta \right)\approx \text{exp}\left\{ - \left(N-k+1\right){\mathcal{E}}_{k}\left(\xi ,\eta \right)\right\} ;\text{ for large }\xi \text{ and }\eta .$$

From Eq. (5), it is seen that the accurate estimation of the bivariate CDF $P(\xi ,\eta )$ is based on an equally accurate estimation of ACER2D functions.

**Figure 10:**
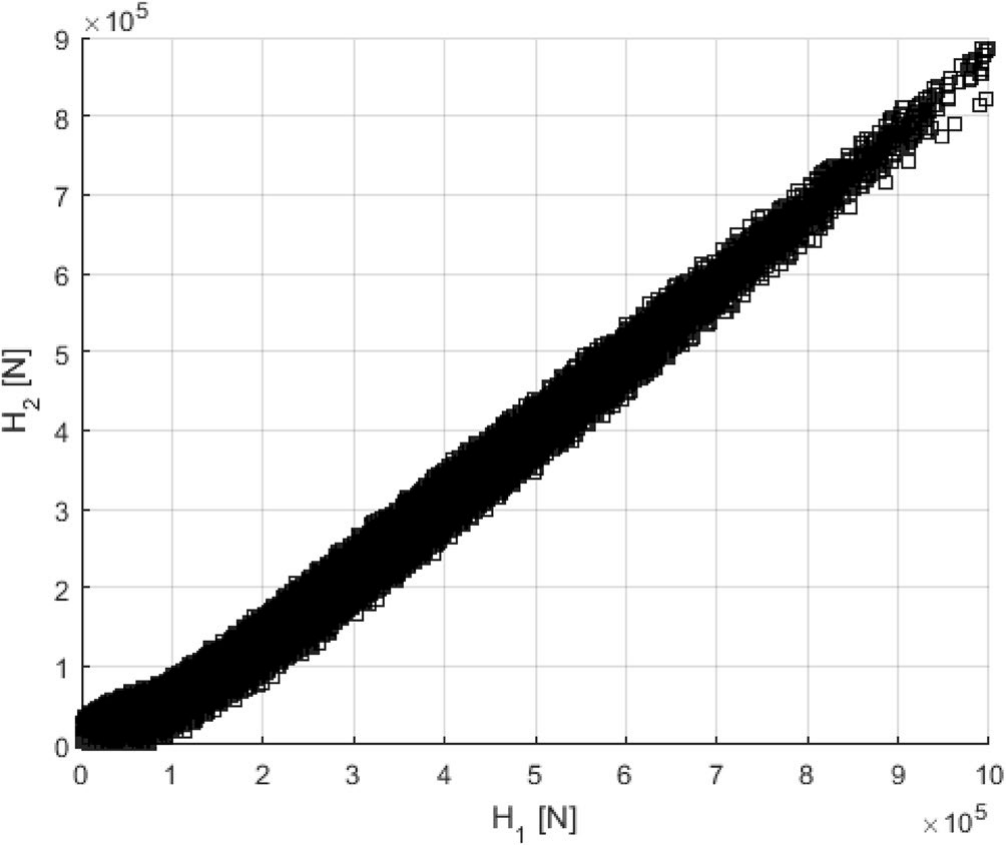
Correlation between neighbouring FPSO stern hawser tensions, H1 versus H2, see Fig. 3.

Figure 10 illustrates the correlation pattern between neighbouring FPSO stern hawser tensions, H1 and H2, in a bivariate plot of the sampled data; see Fig. 3 for H1 and H2 locations. It is clear from Fig. 10 that the H1 and H2 tensions are slightly non-linearly correlated.

By considering the hawser tension PSDs shown in Fig. 6, it can be seen that the tension is characterized by a few narrow-band components. The major component has its natural period of somewhat less than 20 s. Therefore, to observe the dependence effect in the hawser tension time series, the ACER_100_ function should be considered. The latter is because 20 s corresponds to 100 sample points at this paper's 0.2-s discrete time step. The major objective is conditioning above the largest narrow band natural period.

**Figure 11:**
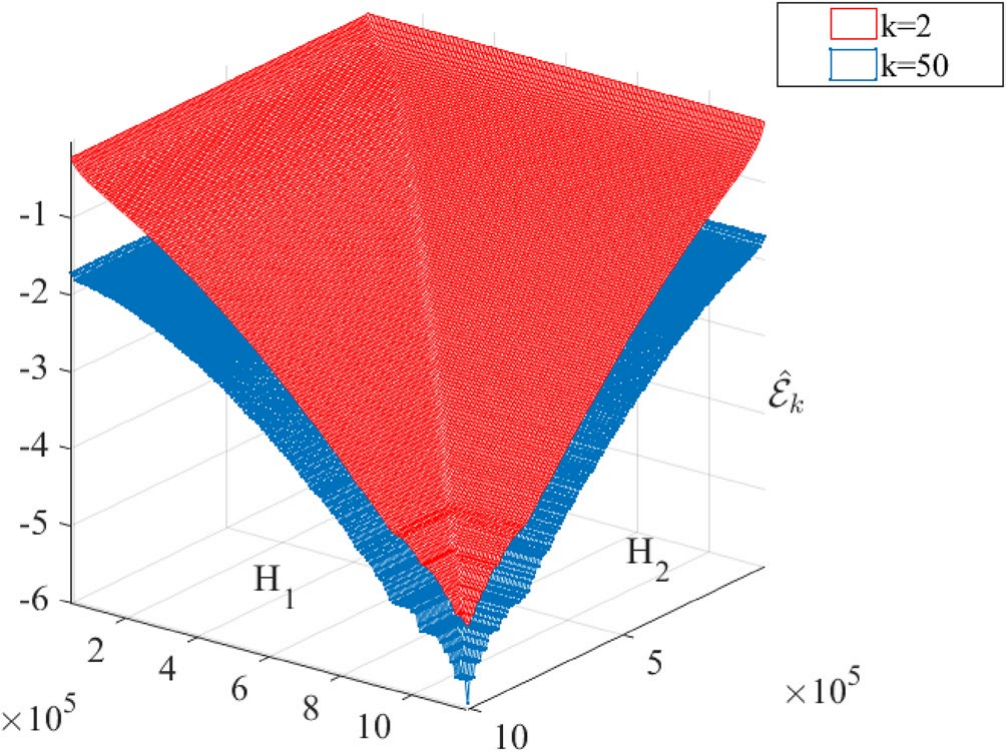
ACER2D surfaces comparison for different conditioning degrees. ${\widehat{\mathcal{E}}}_{k}(\xi ,\eta )$ functions plotted on a decimal logarithmic scale; $\xi$ is H_1_, $\eta$ is H_2_ hawser tension in Newton [N].

**Figure 12:**
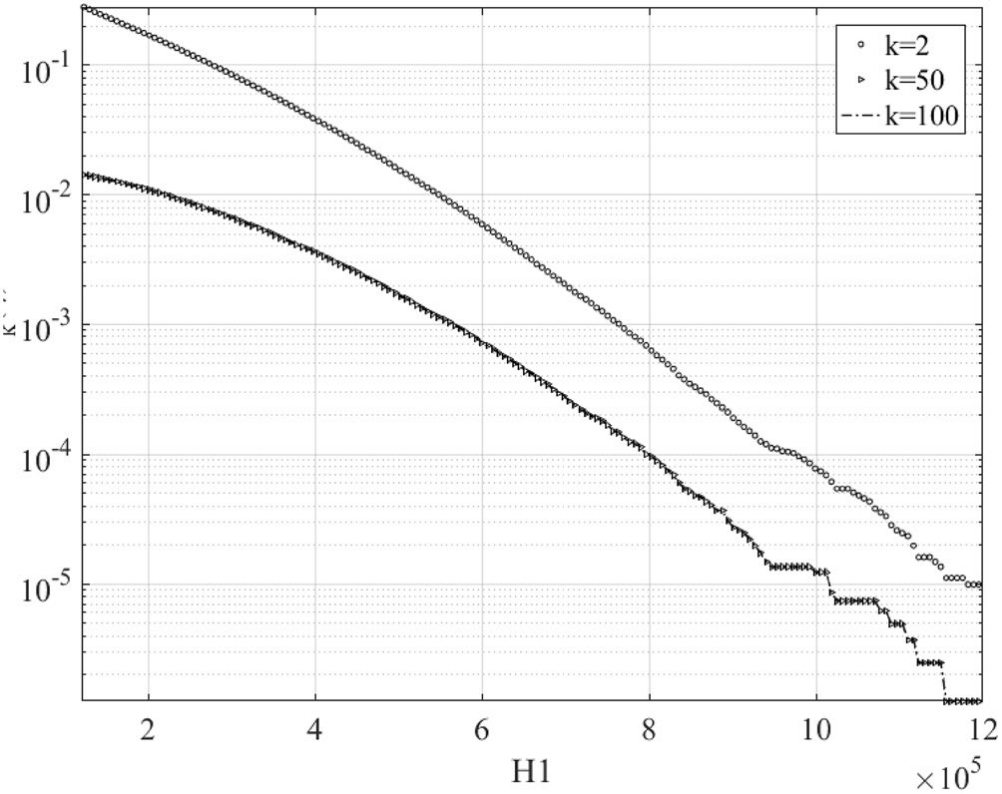
Marginal ACER_k_ functions on the decimal logarithmic scale, $k=\mathrm{2,50,100}$. Note that $k=\mathrm{50,100}$ curves are practically indistinguishable (converged).

Figure 11 presents bivariate ACER2D functions ${\widehat{\mathcal{E}}}_{k}(\xi ,\eta )$ empirically calculated for various conditioning values of $k$ on a decimal logarithmic scale. ${\widehat{\mathcal{E}}}_{k}(\xi ,\eta )$ with $k=2$ is given by the upper surface, while two lower surfaces correspond to $k=50$, note that surface with $k=100$ is not plotted as it is practically indistinguishable from the surface with $k=50$, as seen in Fig. 12, therefore convergence has been achieved. In Fig. 12, marginal ACER_k_ functions are plotted on the decimal logarithmic scale for levels of conditioning $k=2, 50$. It is seen that ACER_2_ significantly deviates from ACER_50_ meaning that there is a strong clustering effect, which has been captured already at the conditioning level $k=50$, since the ACER_100_ function with conditioning level $k=100$ is indistinguishable from the ACER_50_ function with the level of conditioning $k=50$.

**Figure 13:**
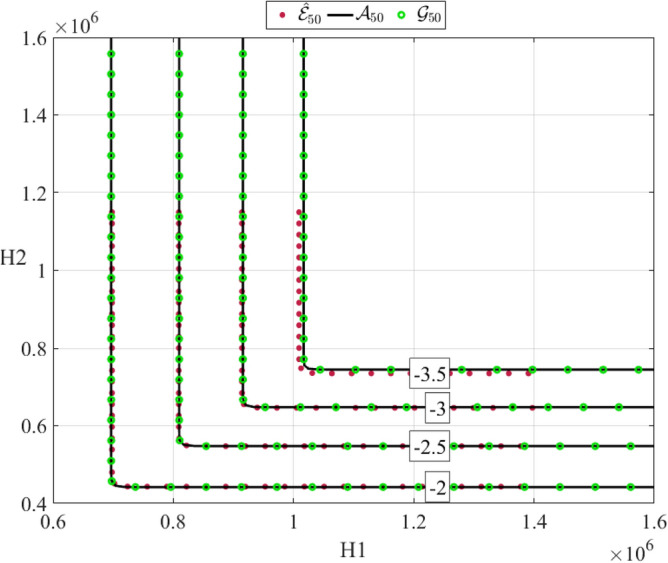
Empirically estimated ${\widehat{\mathcal{E}}}_{50}(\xi ,\eta )$ surface ($\bullet$) contour plot, along with optimized Gumbel logistic ${\mathcal{G}}_{50}(\xi ,\eta )$ ($\circ$) and optimized Asymmetric logistic ${\mathcal{A}}_{50}(\xi ,\eta )$ (–) surfaces. Negative labelling numbers indicate decimal logarithmic scale probability levels.

The lowest probabilities are shown in Fig. 12 as being equal to the value ${N}^{-1}$ with $N$ being the number of equidistant temporal points present in studied time series, Eqs. (3)–(5). Figure 13 presents optimized Asymmetric logistic (AL) ${\mathcal{A}}_{k}(\xi ,\eta )$ and optimized Gumbel logistic (GL) ${\mathcal{G}}_{k}(\xi ,\eta )$ models contour lines, fitted to corresponding empirical bivariate function ${\widehat{\mathcal{E}}}_{k}(\xi ,\eta )$, with $k=50$. Negative numbers marking contour lines in Fig. 13 being probability levels of $P(\xi ,\eta )$ on the decimal logarithmic scale. Figure 13 indicates that empirical bivariate ACER2D surface ${\widehat{\mathcal{E}}}_{50}$ captures well a strong correlation between two response components. Optimized models ${\mathcal{G}}_{50}$ and ${\mathcal{A}}_{50}$ showing smooth contours that match empirical ACER2D contours. Figure 13 shows fine agreement between optimized AL and GL surfaces and corresponding bivariate ACER2D surfaces.

Instead of the pair of uncoupled univariate design points typically used in the industry, bivariate contours provide bivariate design points with the same return period. The latter strategy might result in a multi-dimensional design point that is less conservative, which would result in lower construction costs^33,36^.

## Conclusions

The hydrodynamic functioning of the FPSO during the SBS offloading operation in moderate sea conditions was investigated. Number crunching was used to model the relative dynamic movements between the FPSO and the neighbouring shuttle tanker and the accompanying hawser tensions. Calculated during the frequency-domain analysis were added masses and QTFs. Hawser tensions were estimated in the time-domain analysis to provide long-term data for offloading operations.

Two stern hawser tensions, either simulated or recorded synchronously in time, are the vessel-linked load effects that the bivariate ACER2D approach is used to account for. Using various bivariate copula models, bivariate extreme value distribution low probability contours have been obtained. The approach described offers the following benefits:

- Because model non-linearities are not simplified by the ACER2D method, which is based on a Monte Carlo methodology.
- Different types of connected data can be explored through numerical simulation or measurement.
- Clustering effects might be taken into consideration.

The ACER2D method could provide a simple and efficient way to locate bivariate copula models that are appropriate for the real-world design. The ACER2D approach offers a reliable estimation of the precise bivariate extreme value without directly incorporating asymptotic assumptions, in contrast to numerous methods based on asymptotic assumptions (e.g. Gumbel, Pareto, POT, Weibull). The suggested method may help with vessel parameter optimization, FPSO hawser damage minimization, and FPSO vessel design. Compared to a pair of uncoupled univariate design points with the same return period currently used in the industry, bivariate contours make it easier to choose bivariate design points. With the latter strategy, design vectors are often less conservative, which lowers construction costs. A future potential design expansion of the bivariate strategy might result from multivariate analysis.

## Data Availability

The datasets used and analyzed during the current study are available from the corresponding author on reasonable request.
